# Crystal structure of *rac*-3-[2,3-bis­(phenyl­sulfan­yl)-3*H*-indol-3-yl]propanoic acid

**DOI:** 10.1107/S2056989015020241

**Published:** 2015-10-31

**Authors:** Wayland E. Noland, Christopher D. Brown, Amanda M. Bisel, Andrew K. Schneerer, Kenneth J. Tritch

**Affiliations:** a Department of Chemistry, University of Minnesota, Minneapolis, MN 55455-0431, USA

**Keywords:** crystal structure, Uhle’s ketone, ergot, 3*H*-indole, thio­ation, O—H⋯N hydrogen bond

## Abstract

The title compound, C_23_H_19_NO_2_S_2_, was obtained as an unexpected regioisomer from an attempted synthesis of an inter­mediate for a substituent-effect study on ergot alkaloids. This is the first report of a 1*H*-indole mono­thio­ating at the 2- and 3-positions to give a 3*H*-indole. In the crystal, the acid H atom is twisted roughly 180° from the typical carb­oxy conformation and forms centrosymmetric O—H⋯N hydrogen-bonded dimers with the indole N atom of an inversion-related mol­ecule. Together with a weak C—H⋯O hydrogen bond involving the carbonyl O atom, chains are formed along [100].

## Abstract 2

In the crystal, the acid H atom is twisted roughly 180° from the typical carb­oxy conformation and mol­ecules are linked by pairs of O—H⋯N hydrogen bonds, involving the indole N atom, forming inversion dimers. Together with a weak C—H⋯O hydrogen bond, involving the carbonyl O atom, chains of inversion dimers are formed along [100].

## Chemical context   

**Figure 1:**
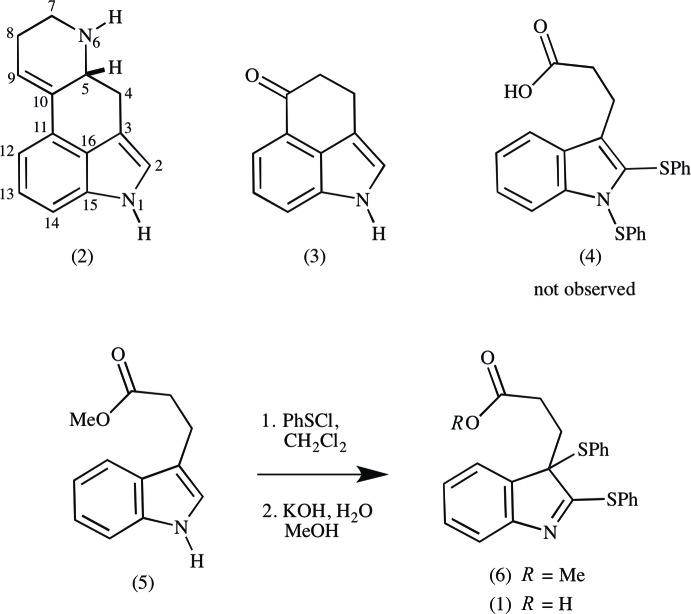
The ergot alkaloid skeleton, (2), Uhle’s ketone, (3), the intended product, (4), and the synthesis of the title compound (bottom row).

The ergot alkaloids, a family of natural and synthetic compounds based on a tetra­cyclic skeleton [(2), Fig. 1 ▸], have been long known to exhibit various pharmacological activities (Hofmann, 1978 ▸). Examples include pergolide (Gilbert *et al.*, 2000 ▸), bromo­criptine (Weber *et al.*, 1981 ▸), and cabergoline (Dosa *et al.*, 2013 ▸), which have been used as treatments for Tourette’s syndrome, psoriasis, and Parkinson’s disease, respectively. Uhle’s ketone (3) is a commonly used inter­mediate in the synthesis of some ergot alkaloids (Moldvai *et al.*, 2004 ▸; Uhle, 1951 ▸).
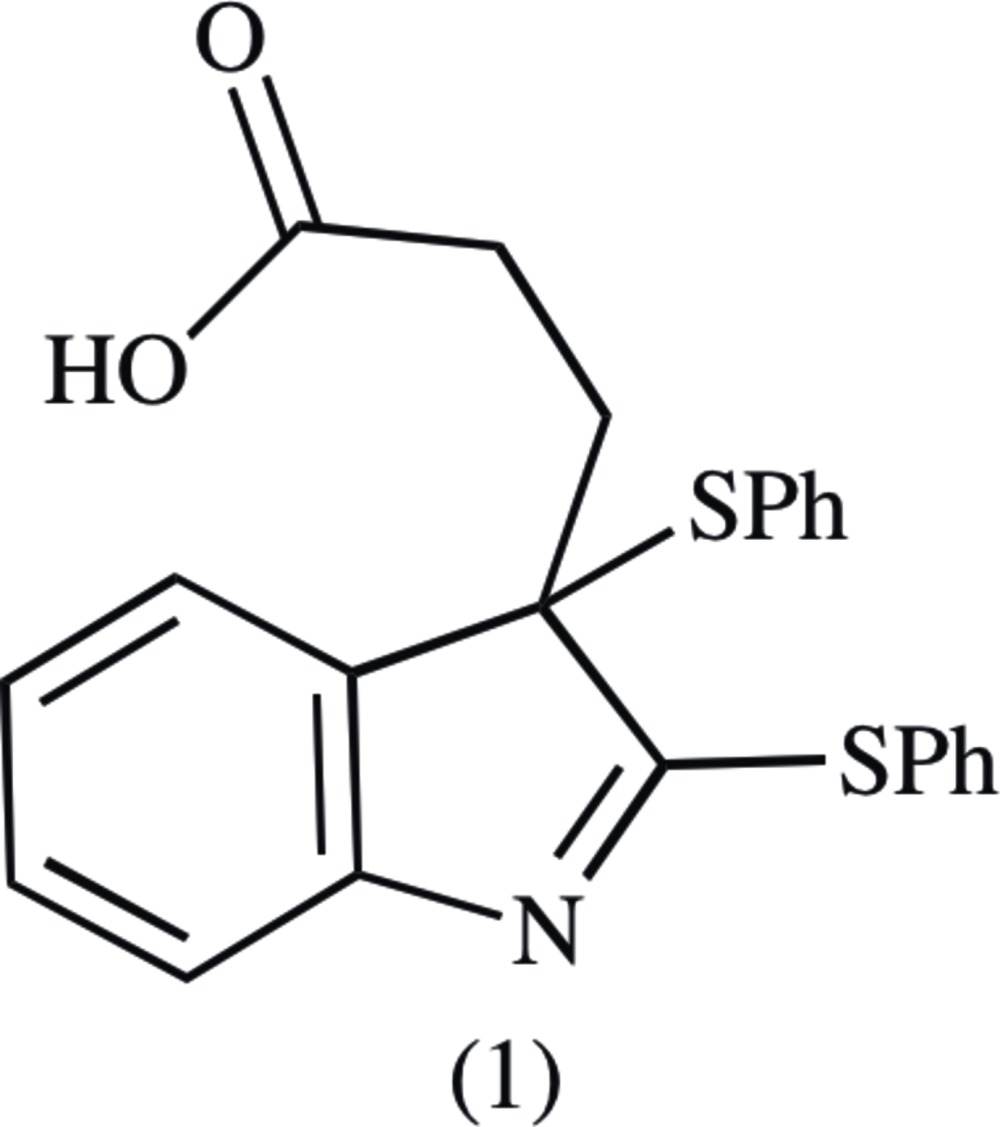


Our group envisioned the synthesis of novel Uhle’s ketone derivatives bearing a reductively removable thio functionality at the 1- or 2-position to facilitate study of substituent effects at the 12–14 positions of several ergot alkaloids. 1,2-Bis(phenyl­thio)­indole-3-propanoic acid (4) was a planned inter­mediate. However, phenyl­thio­ation and hydrolysis of methyl indole-3-propano­ate (5) gave the title compound (1) as the only observed bis­thio­ation product. 2,3-bis­(thio)-3*H*-indoles such as (1) have not previously been reported as a product of 3-alkyl­indoles reacting with sulfenyl chlorides.

## Structural commentary   

**Figure 2:**
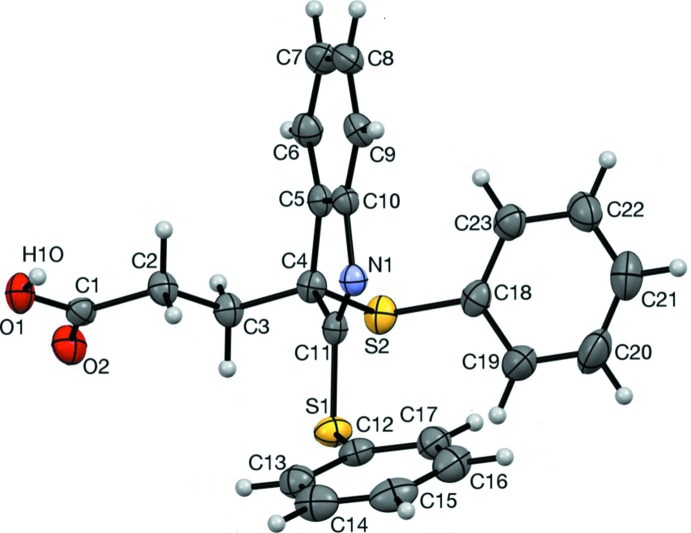
The mol­ecular structure of the title compound, showing the atom labeling. Displacement ellipsoids are drawn at the 50% probability level.

**Figure 3:**
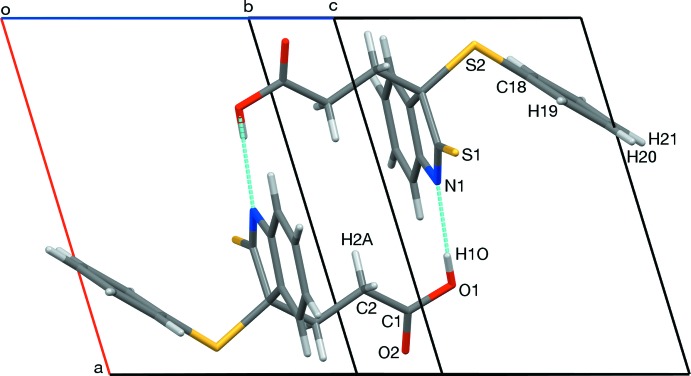
A view along [04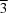] of the O1—H1*O*⋯N1 hydrogen-bonded inversion dimer. The C12–C17 ring has been omitted for clarity.

The mol­ecular structure of the title compound is shown in Fig. 2 ▸. The O1—H1*O* bond is *syn*-periplanar with C1—C2 (Fig. 3 ▸), in contrast to the *anti*-periplanar hydroxyl conformation usually observed in carboxyl groups. This is a consequence of an O1—H1*O*⋯N1 hydrogen bond (Table 1 ▸; §3). The remaining structural features are typical. The atoms of the indole unit (N1/C4–C11) have an r.m.s. deviation of 0.010 (2) Å from the mean plane, with quaternary carbon C4 only 0.012 (2) Å out of plane. The O1/C1–C4/S2/C18 (O1–C18) chain adopts a staggered conformation whose plane of best fit is inclined by 87.97 (8)° to that of the indole unit. Phenyl ring C18–C23 is inclined by 79.39 (10)° to the mean plane of the O1–C18 chain. Phenyl ring C12–C17 ring is inclined by 71.91 (7)° to the mean plane of the indole unit (Fig. 2 ▸). The C12—S1 bond is *syn*-periplanar with bond N1=C11, supporting conjugation between atom S1 and the indole system.

## Supra­molecular features   

**Figure 4:**
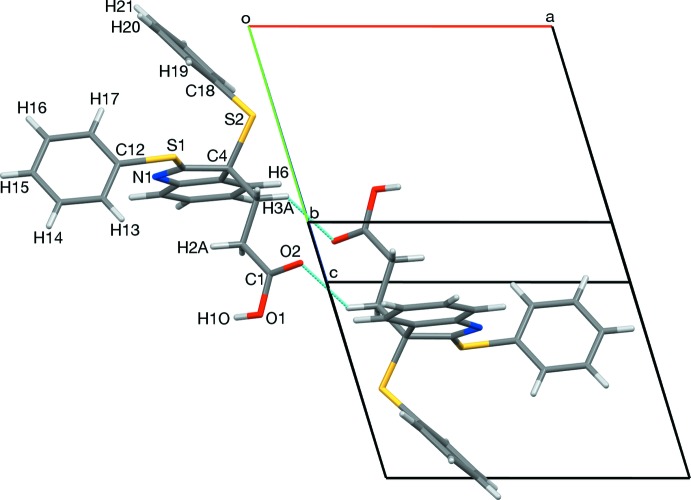
A view along [0
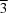
4] of the C3—H3*A*⋯O2 hydrogen-bonded inversion dimer.

In the crystal, an O1—H1*O*⋯N1 hydrogen bond (Table 1 ▸) forms inversion dimers with an 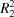
(16) ring motif (Fig. 3 ▸). Mol­ecules are also linked by a non-classical C3—H3*A*⋯O2 hydrogen bond, forming inversion dimers with an 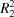
(10) motif (Fig. 4 ▸). Collectively, these inter­actions form chains along [100].

## Database survey   

**Figure 5:**
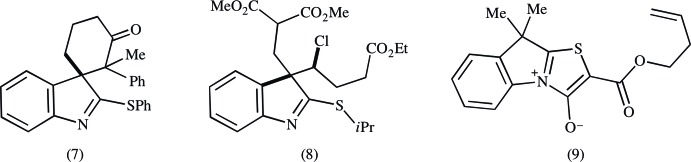
The three 2-thio-3*H*-indoles found in the Cambridge Structural Database (CSD; Groom & Allen, 2014 ▸).

A search was performed for variously substituted 3*H*-indoles in the Cambridge Structural Database (CSD, Version 5.36, update 3; Groom & Allen, 2014 ▸). No entries were found containing a 3-thio or 3-propanoic functionality. Three examples of 2-thio-3*H*-indoles were found. Spiro-fused cyclo­hexa­none (7) contains a 2-phenyl­thio group with similar geometry as is found in the title compound (Fig. 5 ▸; Feldman & Nuriye, 2009 ▸). The long chain in chloro­triester (8) is primarily staggered and normal to the indole unit, akin to the title compound (Novikov *et al.*, 2003 ▸). The third example, (9), is a thia­zolium-4-oxide (Moody *et al.*, 2003 ▸).

## Synthesis and crystallization   

Methyl indole-3-propano­ate (5) was prepared according to Pedras & Jha (2006 ▸), using *p*-toluene­sulfonic acid in place of sulfuric acid. Benzene­sulfenyl chloride (PhSCl) solution was prepared according to Li *et al.* (2013 ▸). In an argon atmosphere, methyl indole-3-propano­ate (2.69 g) was dissolved in di­chloro­methane (30 ml) and then cooled in an ice bath. PhSCl solution (28 mmol, 32 ml) was added dropwise over 30 minutes. The resulting mixture was allowed to warm to room temperature and then was stirred for 2 h. Saturated NaHCO_3_ solution (aq., 30 ml) was added, followed by extraction with di­chloro­methane (3 × 25 ml). The organic portion was dried with MgSO_4_, concentrated, and then purified by column chromatography (SiO_2_, 9:1 hexa­ne–ethyl acetate), giving methyl 2,3-bis­(phenyl­thio)-3*H*-indole-3-propano­ate [(6), *R_f_* = 0.41 in 2:1] as a yellow powder (3.29 g, 59%, m.p. 360-363 K); ^1^H NMR (500 MHz, CD_2_Cl_2_) δ 7.572–7.553 (*m*, 2H), 7.455–7.416 (*m*, 4H), 7.222 (*m*, 1H), 7.194–7.171 (*m*, 4H), 7.104–7.073 (*m*, 2H), 7.045 (*m*, 1H), 3.563 (*s*, 3H), 2.647 (*ddd*, *J* = 13.8, 11.4, 5.1 Hz, 1H), 2.475 (*ddd*, *J* = 13.8, 11.2, 5.1 Hz, 1H), 2.174 (*ddd*, *J* = 16.3, 11.4, 5.1 Hz, 1H), 1.798 (*ddd*, *J* = 16.3, 11.2, 5.1 Hz, 1H); ^13^C NMR (126 MHz, CD_2_Cl_2_) δ 182.70 (1C), 175.84 (1C), 154.68 (1C), 139.60 (1C), 136.18 (2C), 135.44 (2C), 130.06 (1C), 129.95 (1C), 129.79 (2C), 129.74 (1C), 129.42 (1C), 128.85 (2C), 128.57 (1C), 125.31 (1C), 123.62 (1C), 119.50 (1C), 68.10 (1C), 52.14 (1C), 31.92 (1C), 29.55 (1C); IR (KBr, cm^−1^) 3057 (*w*), 2956, 2926, 2851 (*w*), 1734 (*s*, C=O), 1508, 1440 (*s*), 1372, 1298 (O—CH_3_), 1173, 744 (*s*), 689; MS (ESI, *m*/*z*) [*M*+H]^+^ calculated for C_24_H_21_NO_2_S_2_ 420.1086, found 420.1081.

Bisthio­ated ester [(6), 0.52 g] was dissolved in methanol (20 ml). KOH (0.12 g) and water (5 ml) were added. The resulting mixture was refluxed for 1 h and then cooled to room temperature. Hydro­chloric acid was added drop wise until the reaction mixture pH reached 1. The resulting mixture was extracted with di­chloro­methane (3 × 25 ml). The organic portion was dried with MgSO_4_ and then concentrated giving the title compound (1) as a pale-yellow powder (0.43 g, 90%, m.p. 443–445 K); *R_f_* = 0.49 (SiO_2_, 1:1 hexa­ne–ethyl acetate); ^1^H NMR (500 MHz, CD_2_Cl_2_; acid proton H1*O* not observed) δ 7.520–7.389 (*m*, 6H), 7.245–7.168 (*m*, 5H), 7.108–7.078 (*m*, 2H), 7.045 (*m*, 1H), 2.619 (*ddd*, *J* = 13.9, 11.8, 4.7 Hz, H3*A*), 2.447 (*ddd*, *J* = 13.9, 11.0, 5.0 Hz, H3*B*), 2.130 (*ddd*, *J* = 16.4, 11.8, 5.0 Hz, H2*A*), 1.734 (*ddd*, *J* = 16.4, 11.0, 4.7 Hz, H2*B*); ^13^C NMR (126 MHz, CD_2_Cl_2_) δ 183.24 (C11), 175.84 (C1), 154.38 (C10), 139.52 (C5), 136.20 (2C), 135.50 (2C), 130.16 (1C), 130.14 (1C), 129.91 (2C), 129.61 (1C), 128.90 (2C), 128.15 (1C), 127.48 (1C), 125.52 (1C), 123.65 (1C), 119.36 (1C), 67.93 (C4), 31.67 (C3), 29.18 (C2); IR (KBr, cm^−1^) 3407 (O—H), 3056 (*w*), 2925, 2854 (*w*), 1745 (*s*, C=O), 1514, 1383, 746 (*s*), 689; MS (ESI, *m*/*z*) [*M* – H]^−^ calculated for C_23_H_19_NO_2_S_2_ 404.0784, found 404.0797.

Crystals of the title compound were grown by slow evaporation of a solution in di­chloro­methane at 270 K.

## Refinement   

Crystal data, data collection and structure refinement details are summarized in Table 2 ▸. H atoms were placed in calculated positions and refined as riding atoms: O—H = 0.84 Å and C—H = 0.95–0.99 Å with *U*_iso_(H) = 1.5*U*_eq_(O1) for atom H1 and 1.2*U*_eq_(C) for other H atoms.

## Supplementary Material

Crystal structure: contains datablock(s) I. DOI: 10.1107/S2056989015020241/su5230sup1.cif

Structure factors: contains datablock(s) I. DOI: 10.1107/S2056989015020241/su5230Isup2.hkl

Supporting information file. DOI: 10.1107/S2056989015020241/su5230Isup3.cml

CCDC reference: 1433300

Additional supporting information:  crystallographic information; 3D view; checkCIF report

## Figures and Tables

**Table 1:** Hydrogen-bond geometry (, )

*D*H*A*	*D*H	H*A*	*D* *A*	*D*H*A*
O1H1N1^i^	0.84	1.96	2.7622(18)	159
C3H3*A*O2^ii^	0.99	2.57	3.356(2)	136

Symmetry codes: (i) 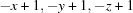; (ii) 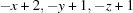.

**Table 2:** Experimental details

Crystal data	
Chemical formula	C_23_H_19_NO_2_S_2_
*M*_r_	405.51
Crystal system, space group	Triclinic, *P* 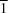
Temperature (K)	173
*a*, *b*, *c* ()	9.6498(12), 9.8610(12), 10.8812(13)
, , ()	87.626(1), 79.331(1), 76.022(1)
*V* (^3^)	987.4(2)
*Z*	2
Radiation type	Mo *K*
(mm^1^)	0.29
Crystal size (mm)	0.23 0.12 0.10

Data collection	
Diffractometer	Bruker APEXII CCD
Absorption correction	Multi-scan (*SADABS*; Bruker, 2007 ▸)
*T*_min_, *T*_max_	0.698, 0.746
No. of measured, independent and observed [*I* > 2(*I*)] reflections	11689, 4499, 3396
*R*_int_	0.032
(sin /)_max_ (^1^)	0.651

Refinement	
*R*[*F* ^2^ > 2(*F* ^2^)], *wR*(*F* ^2^), *S*	0.038, 0.092, 1.06
No. of reflections	4499
No. of parameters	254
H-atom treatment	H-atom parameters constrained
_max_, _min_ (e ^3^)	0.27, 0.26

Computer programs: *APEX2* and *SAINT* (Bruker, 2007 ▸), *SHELXS97* (Sheldrick, 2008 ▸), *SHELXL2014* (Sheldrick, 2015 ▸), *Mercury* (Macrae *et al.*, 2008 ▸), *enCIFer* (Allen *et al.*, 2004 ▸) and *publCIF* (Westrip, 2010 ▸).

## supplementary crystallographic information

### Crystal data

**Table:**

C_23_H_19_NO_2_S_2_	*F*(000) = 424
*M**_r_* = 405.51	*D*_x_ = 1.364 Mg m^−^^3^
Triclinic, *P*1	Melting point: 444 K
*a* = 9.6498 (12) Å	Mo *K*α radiation, λ = 0.71073 Å
*b* = 9.8610 (12) Å	Cell parameters from 2977 reflections
*c* = 10.8812 (13) Å	θ = 2.7–27.3°
α = 87.626 (1)°	µ = 0.29 mm^−^^1^
β = 79.331 (1)°	*T* = 173 K
γ = 76.022 (1)°	Block, colourless
*V* = 987.4 (2) Å^3^	0.23 × 0.12 × 0.10 mm
*Z* = 2	

### Data collection

**Table:**

Bruker APEXII CCD diffractometer	3396 reflections with *I* > 2σ(*I*)
Radiation source: sealed tube	*R*_int_ = 0.032
φ and ω scans	θ_max_ = 27.6°, θ_min_ = 1.9°
Absorption correction: multi-scan (*SADABS*; Bruker, 2007)	*h* = −12→12
*T*_min_ = 0.698, *T*_max_ = 0.746	*k* = −12→12
11689 measured reflections	*l* = −14→14
4499 independent reflections	

### Refinement

**Table:**

Refinement on *F*^2^	0 restraints
Least-squares matrix: full	Hydrogen site location: inferred from neighbouring sites
*R*[*F*^2^ > 2σ(*F*^2^)] = 0.038	H-atom parameters constrained
*wR*(*F*^2^) = 0.092	*w* = 1/[σ^2^(*F*_o_^2^) + (0.0362*P*)^2^ + 0.1618*P*] where *P* = (*F*_o_^2^ + 2*F*_c_^2^)/3
*S* = 1.06	(Δ/σ)_max_ < 0.001
4499 reflections	Δρ_max_ = 0.27 e Å^−^^3^
254 parameters	Δρ_min_ = −0.26 e Å^−^^3^

### Special details

**Table:**

Geometry. All e.s.d.'s (except the e.s.d. in the dihedral angle between two l.s. planes) are estimated using the full covariance matrix. The cell e.s.d.'s are taken into account individually in the estimation of e.s.d.'s in distances, angles and torsion angles; correlations between e.s.d.'s in cell parameters are only used when they are defined by crystal symmetry. An approximate (isotropic) treatment of cell e.s.d.'s is used for estimating e.s.d.'s involving l.s. planes.

### Fractional atomic coordinates and isotropic or equivalent isotropic displacement parameters (Å^2^)

**Table:**

	*x*	*y*	*z*	*U*_iso_*/*U*_eq_	
S1	0.62197 (5)	0.45034 (5)	0.16673 (4)	0.03266 (13)	
S2	0.92472 (5)	0.19331 (5)	0.20164 (4)	0.03283 (13)	
O1	0.74866 (13)	0.71335 (13)	0.58120 (12)	0.0338 (3)	
H1	0.6688	0.6928	0.6075	0.051*	
O2	0.93299 (13)	0.65100 (13)	0.42699 (12)	0.0370 (3)	
N1	0.54112 (14)	0.29215 (14)	0.36472 (13)	0.0251 (3)	
C1	0.82262 (18)	0.62905 (17)	0.48697 (16)	0.0262 (4)	
C2	0.76080 (18)	0.50855 (18)	0.46281 (16)	0.0289 (4)	
H2A	0.6624	0.5453	0.4433	0.035*	
H2B	0.7514	0.4515	0.5395	0.035*	
C3	0.85486 (18)	0.41595 (17)	0.35510 (16)	0.0279 (4)	
H3A	0.9532	0.3789	0.3747	0.033*	
H3B	0.8644	0.4731	0.2784	0.033*	
C4	0.79162 (17)	0.29341 (17)	0.33029 (15)	0.0258 (4)	
C5	0.75579 (18)	0.20556 (17)	0.44228 (15)	0.0254 (4)	
C6	0.84042 (19)	0.13254 (18)	0.52368 (17)	0.0310 (4)	
H6	0.9401	0.1332	0.5147	0.037*	
C7	0.7755 (2)	0.05780 (18)	0.61945 (17)	0.0350 (4)	
H7	0.8320	0.0052	0.6757	0.042*	
C8	0.6292 (2)	0.05935 (18)	0.63364 (17)	0.0350 (4)	
H8	0.5872	0.0073	0.6994	0.042*	
C9	0.54286 (19)	0.13564 (17)	0.55330 (16)	0.0295 (4)	
H9	0.4424	0.1380	0.5637	0.035*	
C10	0.60945 (18)	0.20787 (16)	0.45758 (15)	0.0251 (4)	
C11	0.64132 (17)	0.34029 (17)	0.29370 (15)	0.0250 (4)	
C12	0.43242 (19)	0.48511 (19)	0.16692 (15)	0.0295 (4)	
C13	0.3474 (2)	0.6178 (2)	0.20041 (17)	0.0363 (4)	
H13	0.3887	0.6865	0.2283	0.044*	
C14	0.2017 (2)	0.6485 (2)	0.19258 (18)	0.0432 (5)	
H14	0.1427	0.7391	0.2152	0.052*	
C15	0.1417 (2)	0.5497 (2)	0.15248 (18)	0.0442 (5)	
H15	0.0420	0.5726	0.1459	0.053*	
C16	0.2255 (2)	0.4172 (2)	0.12167 (18)	0.0422 (5)	
H16	0.1828	0.3482	0.0962	0.051*	
C17	0.3717 (2)	0.3847 (2)	0.12777 (17)	0.0358 (4)	
H17	0.4301	0.2939	0.1052	0.043*	
C18	0.83442 (18)	0.06781 (19)	0.16376 (16)	0.0307 (4)	
C19	0.7666 (2)	0.0890 (2)	0.06019 (18)	0.0444 (5)	
H19	0.7669	0.1712	0.0115	0.053*	
C20	0.6988 (3)	−0.0091 (3)	0.0277 (2)	0.0563 (6)	
H20	0.6528	0.0057	−0.0433	0.068*	
C21	0.6979 (2)	−0.1282 (2)	0.0980 (2)	0.0506 (6)	
H21	0.6496	−0.1947	0.0764	0.061*	
C22	0.7667 (2)	−0.1509 (2)	0.19934 (19)	0.0444 (5)	
H22	0.7674	−0.2341	0.2467	0.053*	
C23	0.8348 (2)	−0.05393 (19)	0.23299 (17)	0.0355 (4)	
H23	0.8820	−0.0703	0.3034	0.043*	

### Atomic displacement parameters (Å^2^)

**Table:**

	*U*^11^	*U*^22^	*U*^33^	*U*^12^	*U*^13^	*U*^23^
S1	0.0282 (2)	0.0393 (3)	0.0309 (2)	−0.0096 (2)	−0.00598 (19)	0.0068 (2)
S2	0.0249 (2)	0.0364 (3)	0.0345 (3)	−0.00567 (19)	0.00119 (19)	−0.0076 (2)
O1	0.0281 (7)	0.0349 (7)	0.0405 (7)	−0.0121 (6)	−0.0035 (6)	−0.0088 (6)
O2	0.0303 (7)	0.0405 (8)	0.0426 (8)	−0.0175 (6)	0.0000 (6)	−0.0020 (6)
N1	0.0236 (7)	0.0249 (7)	0.0271 (7)	−0.0060 (6)	−0.0048 (6)	−0.0018 (6)
C1	0.0255 (9)	0.0273 (9)	0.0282 (9)	−0.0072 (7)	−0.0101 (7)	0.0035 (7)
C2	0.0246 (9)	0.0300 (9)	0.0326 (9)	−0.0104 (7)	−0.0011 (7)	−0.0025 (7)
C3	0.0223 (8)	0.0299 (9)	0.0325 (9)	−0.0089 (7)	−0.0038 (7)	−0.0017 (7)
C4	0.0208 (8)	0.0271 (9)	0.0279 (9)	−0.0035 (7)	−0.0027 (7)	−0.0034 (7)
C5	0.0262 (9)	0.0216 (8)	0.0274 (9)	−0.0041 (7)	−0.0033 (7)	−0.0047 (7)
C6	0.0290 (9)	0.0268 (9)	0.0361 (10)	−0.0031 (7)	−0.0075 (8)	−0.0026 (8)
C7	0.0397 (11)	0.0265 (9)	0.0368 (11)	0.0001 (8)	−0.0127 (8)	0.0012 (8)
C8	0.0449 (11)	0.0254 (9)	0.0337 (10)	−0.0089 (8)	−0.0050 (9)	0.0037 (8)
C9	0.0287 (9)	0.0239 (9)	0.0362 (10)	−0.0082 (7)	−0.0042 (8)	−0.0006 (7)
C10	0.0273 (9)	0.0212 (8)	0.0265 (9)	−0.0048 (7)	−0.0046 (7)	−0.0024 (7)
C11	0.0241 (8)	0.0231 (8)	0.0270 (9)	−0.0040 (7)	−0.0036 (7)	−0.0065 (7)
C12	0.0291 (9)	0.0360 (10)	0.0223 (9)	−0.0058 (8)	−0.0054 (7)	0.0048 (7)
C13	0.0371 (10)	0.0378 (11)	0.0320 (10)	−0.0065 (8)	−0.0043 (8)	0.0006 (8)
C14	0.0363 (11)	0.0459 (12)	0.0380 (11)	0.0020 (9)	0.0014 (9)	0.0029 (9)
C15	0.0280 (10)	0.0677 (15)	0.0333 (11)	−0.0099 (10)	−0.0007 (8)	0.0100 (10)
C16	0.0385 (11)	0.0565 (13)	0.0360 (11)	−0.0199 (10)	−0.0072 (9)	0.0033 (10)
C17	0.0376 (11)	0.0377 (11)	0.0327 (10)	−0.0090 (9)	−0.0075 (8)	0.0004 (8)
C18	0.0264 (9)	0.0330 (10)	0.0291 (9)	−0.0006 (7)	−0.0022 (7)	−0.0085 (8)
C19	0.0580 (14)	0.0411 (12)	0.0346 (11)	−0.0078 (10)	−0.0146 (10)	−0.0018 (9)
C20	0.0702 (16)	0.0599 (15)	0.0451 (13)	−0.0130 (12)	−0.0271 (12)	−0.0136 (11)
C21	0.0531 (14)	0.0479 (13)	0.0542 (14)	−0.0160 (11)	−0.0092 (11)	−0.0176 (11)
C22	0.0538 (13)	0.0347 (11)	0.0425 (12)	−0.0113 (10)	−0.0007 (10)	−0.0057 (9)
C23	0.0374 (10)	0.0328 (10)	0.0335 (10)	−0.0013 (8)	−0.0072 (8)	−0.0050 (8)

### Geometric parameters (Å, º)

**Table:**

S1—C11	1.7305 (17)	C8—H8	0.9500
S1—C12	1.7773 (18)	C9—C10	1.385 (2)
S2—C18	1.7767 (18)	C9—H9	0.9500
S2—C4	1.8493 (16)	C12—C17	1.383 (2)
O1—C1	1.328 (2)	C12—C13	1.389 (3)
O1—H1	0.8400	C13—C14	1.382 (3)
O2—C1	1.2040 (19)	C13—H13	0.9500
N1—C11	1.293 (2)	C14—C15	1.370 (3)
N1—C10	1.435 (2)	C14—H14	0.9500
C1—C2	1.504 (2)	C15—C16	1.378 (3)
C2—C3	1.525 (2)	C15—H15	0.9500
C2—H2A	0.9900	C16—C17	1.382 (3)
C2—H2B	0.9900	C16—H16	0.9500
C3—C4	1.533 (2)	C17—H17	0.9500
C3—H3A	0.9900	C18—C19	1.389 (3)
C3—H3B	0.9900	C18—C23	1.390 (2)
C4—C5	1.502 (2)	C19—C20	1.382 (3)
C4—C11	1.533 (2)	C19—H19	0.9500
C5—C6	1.379 (2)	C20—C21	1.375 (3)
C5—C10	1.386 (2)	C20—H20	0.9500
C6—C7	1.392 (2)	C21—C22	1.374 (3)
C6—H6	0.9500	C21—H21	0.9500
C7—C8	1.388 (3)	C22—C23	1.379 (3)
C7—H7	0.9500	C22—H22	0.9500
C8—C9	1.392 (2)	C23—H23	0.9500
			
C11—S1—C12	102.47 (8)	C9—C10—N1	126.35 (15)
C18—S2—C4	102.65 (8)	C5—C10—N1	111.96 (14)
C1—O1—H1	109.5	N1—C11—C4	114.43 (14)
C11—N1—C10	106.38 (14)	N1—C11—S1	127.16 (13)
O2—C1—O1	119.92 (15)	C4—C11—S1	118.40 (12)
O2—C1—C2	123.81 (16)	C17—C12—C13	120.40 (17)
O1—C1—C2	116.27 (14)	C17—C12—S1	120.78 (14)
C1—C2—C3	112.44 (13)	C13—C12—S1	118.72 (14)
C1—C2—H2A	109.1	C14—C13—C12	119.08 (19)
C3—C2—H2A	109.1	C14—C13—H13	120.5
C1—C2—H2B	109.1	C12—C13—H13	120.5
C3—C2—H2B	109.1	C15—C14—C13	120.61 (19)
H2A—C2—H2B	107.8	C15—C14—H14	119.7
C2—C3—C4	112.42 (13)	C13—C14—H14	119.7
C2—C3—H3A	109.1	C14—C15—C16	120.29 (19)
C4—C3—H3A	109.1	C14—C15—H15	119.9
C2—C3—H3B	109.1	C16—C15—H15	119.9
C4—C3—H3B	109.1	C15—C16—C17	119.99 (19)
H3A—C3—H3B	107.9	C15—C16—H16	120.0
C5—C4—C3	115.35 (14)	C17—C16—H16	120.0
C5—C4—C11	99.58 (13)	C16—C17—C12	119.61 (18)
C3—C4—C11	113.08 (13)	C16—C17—H17	120.2
C5—C4—S2	113.36 (11)	C12—C17—H17	120.2
C3—C4—S2	104.98 (11)	C19—C18—C23	119.20 (18)
C11—C4—S2	110.69 (11)	C19—C18—S2	119.45 (15)
C6—C5—C10	120.95 (16)	C23—C18—S2	121.31 (14)
C6—C5—C4	131.39 (15)	C20—C19—C18	120.20 (19)
C10—C5—C4	107.65 (14)	C20—C19—H19	119.9
C5—C6—C7	118.04 (17)	C18—C19—H19	119.9
C5—C6—H6	121.0	C21—C20—C19	120.1 (2)
C7—C6—H6	121.0	C21—C20—H20	119.9
C8—C7—C6	120.76 (17)	C19—C20—H20	119.9
C8—C7—H7	119.6	C22—C21—C20	119.9 (2)
C6—C7—H7	119.6	C22—C21—H21	120.0
C7—C8—C9	121.26 (17)	C20—C21—H21	120.0
C7—C8—H8	119.4	C21—C22—C23	120.6 (2)
C9—C8—H8	119.4	C21—C22—H22	119.7
C10—C9—C8	117.26 (16)	C23—C22—H22	119.7
C10—C9—H9	121.4	C22—C23—C18	119.89 (18)
C8—C9—H9	121.4	C22—C23—H23	120.1
C9—C10—C5	121.70 (16)	C18—C23—H23	120.1
			
O2—C1—C2—C3	0.9 (2)	C10—N1—C11—S1	−179.11 (12)
O1—C1—C2—C3	−178.65 (14)	C5—C4—C11—N1	−0.43 (18)
C1—C2—C3—C4	−179.85 (13)	C3—C4—C11—N1	−123.38 (16)
C2—C3—C4—C5	−52.58 (19)	S2—C4—C11—N1	119.15 (13)
C2—C3—C4—C11	61.13 (19)	C5—C4—C11—S1	178.90 (11)
C2—C3—C4—S2	−178.10 (12)	C3—C4—C11—S1	55.95 (17)
C18—S2—C4—C5	60.67 (13)	S2—C4—C11—S1	−61.52 (14)
C18—S2—C4—C3	−172.58 (11)	C12—S1—C11—N1	3.10 (17)
C18—S2—C4—C11	−50.24 (13)	C12—S1—C11—C4	−176.13 (12)
C3—C4—C5—C6	−57.2 (2)	C11—S1—C12—C17	−74.16 (15)
C11—C4—C5—C6	−178.58 (17)	C11—S1—C12—C13	109.44 (14)
S2—C4—C5—C6	63.8 (2)	C17—C12—C13—C14	−0.8 (3)
C3—C4—C5—C10	121.86 (15)	S1—C12—C13—C14	175.62 (14)
C11—C4—C5—C10	0.53 (16)	C12—C13—C14—C15	0.1 (3)
S2—C4—C5—C10	−117.07 (13)	C13—C14—C15—C16	1.3 (3)
C10—C5—C6—C7	1.9 (2)	C14—C15—C16—C17	−1.8 (3)
C4—C5—C6—C7	−179.11 (16)	C15—C16—C17—C12	1.1 (3)
C5—C6—C7—C8	−1.2 (3)	C13—C12—C17—C16	0.2 (3)
C6—C7—C8—C9	−0.2 (3)	S1—C12—C17—C16	−176.11 (14)
C7—C8—C9—C10	1.0 (3)	C4—S2—C18—C19	100.86 (16)
C8—C9—C10—C5	−0.4 (2)	C4—S2—C18—C23	−81.59 (15)
C8—C9—C10—N1	179.83 (15)	C23—C18—C19—C20	0.9 (3)
C6—C5—C10—C9	−1.1 (3)	S2—C18—C19—C20	178.48 (16)
C4—C5—C10—C9	179.70 (15)	C18—C19—C20—C21	0.2 (3)
C6—C5—C10—N1	178.71 (14)	C19—C20—C21—C22	−1.2 (3)
C4—C5—C10—N1	−0.51 (18)	C20—C21—C22—C23	1.2 (3)
C11—N1—C10—C9	−179.99 (16)	C21—C22—C23—C18	−0.2 (3)
C11—N1—C10—C5	0.23 (18)	C19—C18—C23—C22	−0.9 (3)
C10—N1—C11—C4	0.15 (18)	S2—C18—C23—C22	−178.43 (14)

### Hydrogen-bond geometry (Å, º)

**Table:**

*D*—H···*A*	*D*—H	H···*A*	*D*···*A*	*D*—H···*A*
O1—H1···N1^i^	0.84	1.96	2.7622 (18)	159
C3—H3*A*···O2^ii^	0.99	2.57	3.356 (2)	136

Symmetry codes: (i) −*x*+1, −*y*+1, −*z*+1; (ii) −*x*+2, −*y*+1, −*z*+1.
